# Impact of Soil Moisture Dynamics on ASAR *σ*^o^ Signatures and Its Spatial Variability Observed over the Tibetan Plateau

**DOI:** 10.3390/s8095479

**Published:** 2008-09-03

**Authors:** Rogier van der Velde, Zhongbo Su, Yaoming Ma

**Affiliations:** 1 International Institute for Geo-Information Science and Earth Observation (ITC), Hengelosestraat 99, P.O. Box 6, 7500 AA Enschede, The Netherlands; E-mail: b_su@itc.nl (B.Z.S.); 2 Institute of Tibetan Plateau Research (ITP/CAS), P.O. Box 2871, Beijing 100085, P.R. China; E-mail: ymma@itpcas.ac.cn (Y.M.)

**Keywords:** Soil moisture dynamics, ASAR, Time series, Tibetan Plateau

## Abstract

This paper reports on the analysis of a 2.5 year-long time series of ASAR wide swath mode (WSM) observations for characterizing the soil moisture dynamics. The employed ASAR WSM data set consists of 152 VV-polarized scenes acquired in the period between April 2005 and September 2007 over the Naqu river basin located on the Tibetan Plateau. For four different spatial domains, with areas of 30×30 km^2^, 5×5 km^2^ and (two domains of) 1×1 km^2^, the mean backscatter (*σ*^o^) and the standard deviation (stdev) have been computed for each ASAR acquisition. Comparison of the mean *σ*^o^ values with the stdev values results in a specific triangular distribution of data points for all spatial domains. Analysis of the mean *σ*^o^ and stdev with respect to in-situ soil moisture measurements demonstrates that this triangular shaped distribution can be explained by soil moisture dynamics during monsoon and winter periods. This shows that the relationship between the spatial mean soil moisture and variability is not uniquely defined and may change throughout seasons. Downscaling of coarse resolution soil moisture products should, therefore, be ideally based on additional near real time data sources. In this context, the presented results could form a basis for the development of SAR-based soil moisture downscaling methodologies.

## 1. Introduction

The importance of regional-scale spatial and temporal soil moisture dynamics in the development of weather systems has been acknowledged [1-3]. Satellite missions that accommodate the monitoring of this land surface property are currently operational (e.g. Advanced Microwave Scanning Radiometer (AMSR), Advanced Scatterometer (ASCAT), being prepared for launch (e.g. Soil Moisture and Ocean Salinity (SMOS)) and are being formulated (e.g. Soil Moisture Active/Passive (SMAP). Monitoring of the temporal evolutions will be accommodated, but the variability within the radiometer and scatterometer footprints remains difficult to obtain.

Previous investigations [4-6] have focused on describing large-scale soil moisture distributions through statistical spatial analysis of comprehensive in-situ data sets. The diversity in results from those field campaigns indicates that the relationship between statistical moments characterizing the temporal evolution and the spatial variability is not uniquely defined across landscapes and throughout time. For example, Famiglietti *et al.* [7] observed that the mean soil moisture and its variability are negatively correlated, while other investigations [8, 9] have reported on positive correlations. More recently, Ryu and Famiglietti [10] concluded that the relationship between the mean soil moisture and its variability depends on the modality of soil moisture probability density functions (PDFs).

Therefore, determination of the soil moisture distributions within large-scale passive microwave satellite footprints would, ideally, be obtained from additional data sources. High resolution active microwave observations acquired from space through the Synthetic Aperture Radar (SAR) technique have been shown to be sensitive to soil moisture changes [11-13] and could be a good candidate. Although many scientists [14-18] have shown that SAR observations can be utilized to retrieve soil moisture under controlled conditions, the development of operational retrieval methodologies has been less successful for various reasons.

At the spatial scale of SAR observations, variations in surface roughness and vegetation affecting the backscatter (*σ*^o^) are large. Representative parameterizations required to eliminate surface roughness and vegetation effects are difficult to define and imposes large uncertainties on retrievals. Moreover, the temporal resolution of SAR observations is relatively low because of either limitations of the SAR sensors itself (i.e. European Remote Sensing (ERS) satellite -1/2) or conflicts with other users in case of multi mode SAR sensors (e.g. Advanced SAR (ASAR), Phased Array type L-band SAR (PALSAR)). Long-term SAR data sets with the temporal resolution required to capture the dynamics of highly variable land surface states (such as soil moisture conditions) are, therefore, difficult to obtain.

Through consistent data requests in the ESA - MOST (European Space Agency – Ministry of Science and technology, China) – Dragon programme, a 2.5 years long time series of SAR observations has been obtained from ASAR in the wide swath mode (WSM) over the Naqu river basin located on the central part of Tibetan Plateau. This data set includes 152 scenes acquired in the period between April 2005 and September 2007 with an averaged temporal resolution of 6 days. In this paper, this time series is analyzed to study the influence of soil moisture dynamics throughout the selected period on *σ*^o^ signatures and their effect on the spatial *σ*^o^ variability over different spatial domains. Through this analysis the potential of SAR observations to provide information on the soil moisture conditions over aggregated spatial domains is demonstrated, which may form a basis for the development of SAR based methodologies to characterize the spatial variability within coarse resolution microwave radiometer and scatterometer footprints.

## 2. ASAR WSM data sets

The ASAR WSM observations have been requested in the VV-polarization covering a 15-45 degrees view angle range and delivered as ellipsoid geocoded level 1b products with a grid spacing of 75 meters. The data set includes 102 scenes in an ascending orbit and 50 scenes in a descending orbit in the period between April 2005 and September 2007 with 6-day temporal resolution on average. Prior to derivation of the *σ*^o^ observations, the ascending and descending scenes have been separately co-located and the terrain elevation angle has been determined for the ascending and descending view geometries based on the 90 meter resolution Digital Elevation Model (DEM) by the Shuttle Radar Topography Mission (SRTM). Radiometrically terrain corrected *σ*^o^ observations have been derived following ASAR product handbook (available at: http://envisat.esa.int/handbooks/asar, verified on August 18, 2008) using the terrain correction incidence angle. Because the requested WSM product has been processed to 21-look images (3 looks in the azimuth and 7 looks in the range direction), no additional speckle filtering has been applied.

The obtained *σ*^o^ observations have been normalized to an incidence angle of 23 degrees using:

(1) $$\sigma^{o}(23)=\frac{\sigma_{\theta_{i}}^{o}}{\cos^{2}\theta_{i}}$$

where $\sigma_{\theta_{i}}^{o}$ is the ASAR *σ*^o^ observation *σ*^o^ (23) is the backscatter normalized to an incidence angle of 23 degrees and *θ*_i_ is the incidence angle.

This approach is based on Lambert's law for optics, which assumes that the relationship between the incidence angle and amount of scattering per unit surface area follows the cosine law. This behavior is typically observed over the middle range of incidence angles [22], in which ASAR WSM observations have been acquired.

## 3. Description of the study area

In the Naqu river basin a meso-scale network of meteorological stations has been installed in the framework of the GEWEX* sponsored GAME**/Tibet and CAMP***/Tibet field campaigns, of which Naqu station is equipped with the most extensive set of field instruments (e.g. radiation and eddy correlation instrumentation). This station is located near Naqu city at a latitude and longitude of 31.36 and 91.89 degrees (WSG84), respectively. The Naqu station and its vicinity have been selected as the focal point for this study.

For this investigation the top 4-cm soil moisture measured at Naqu station are used. These soil moisture measurements have been recorded using a 10-cm long impedance probe (type: ECH_2_O EC-10) manufactured by Decagon Devices. The probe readings have been calibrated using volumetric soil moisture measurements obtained through gravimetric sampling. After calibration the Root Mean Square Difference (RMSD) between calibrated impedance probe and gravimetrically determined soil moisture is found to be 0.024 [cm^3^cm^-3^].

**Figure 1.**
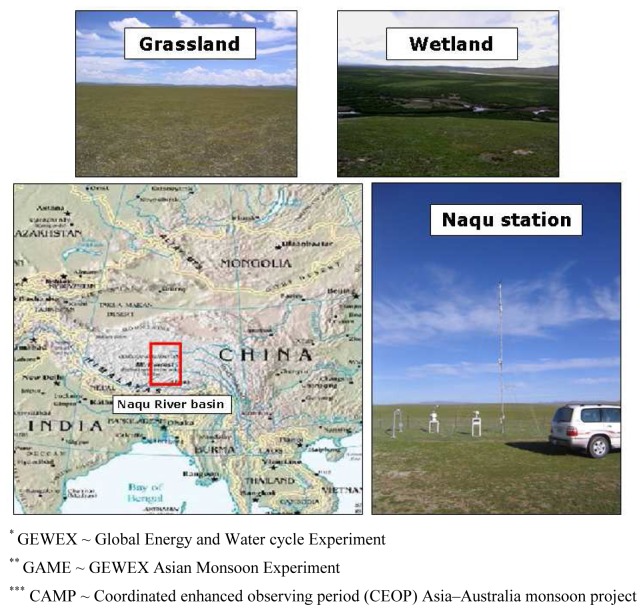
Geographical location of Naqu river basin and photos of Naqu station and a typical grassland and wetland in the study area.

In spite of the overall high altitude, on average 4,500 meters above sea level, the terrain is relatively smooth with rolling hills varying tens of meters in elevation. The top soils have a high saturated hydraulic conductivity (K_sat_ = 1.2 m d^-1^) positioned on top of an impermeable rock formation. Precipitation is, therefore, not able to drain deeply into the ground and will runoff towards the lower parts in the landscape. In these local depressions, wetland vegetation is the dominant land cover. The higher parts are covered by sparse vegetation, which consist of grasses and mosses. Soils in the wetlands have high organic matter content and can be classified as peat, while in the grasslands soils are sandy. Figure 1 shows the geographical location of Naqu river basin and gives an impression of the Naqu station and a typical grassland and wetland in the study area.

The weather in this part of the plateau is influenced by the warm monsoon in the summer and cold dry winters with temperatures below freezing point. During the winter, the soil surfaces of the grasslands as well as wetlands contain small amounts of moisture and are often frozen. During the summer months, surface conditions in the wetlands are predominantly wet due to accumulation of runoff, while soil moisture dynamics over the grassland are highly variable due to processes, such as precipitation, evaporation and transpiration.

## 4. Observed relationships between the mean backscatter and its spatial variability over different domains

**Figure 2.**
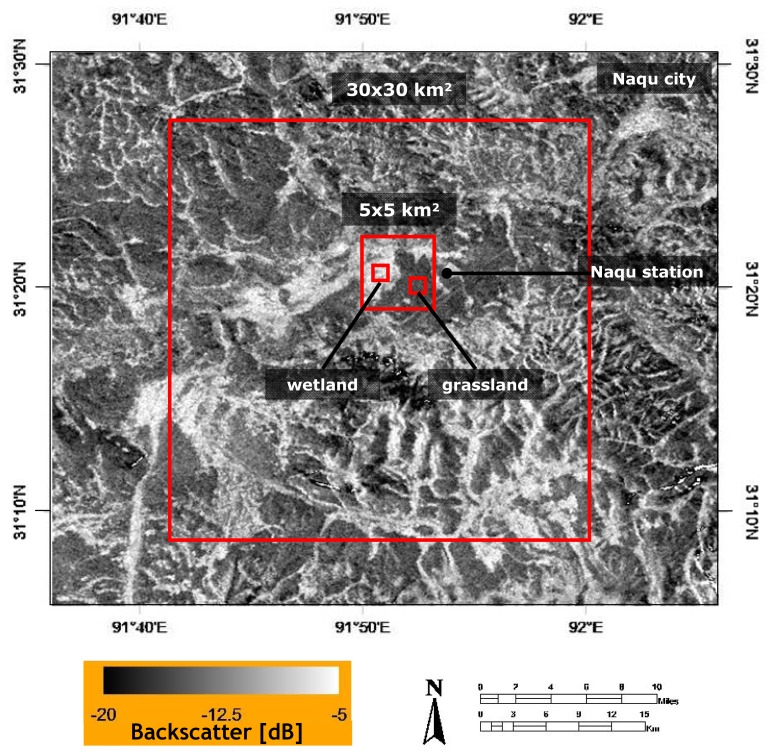
Positioning of the selected 30×30 km^2^, 5×5 km^2^ and 1×1 km^2^ wetland and grassland domains with the study area and the location of Naqu station.

Differences in the land cover and seasonal weather cause soil moisture conditions to be spatially and temporally variable in the area around Naqu station. To investigate the impact of these soil moisture dynamics on *σ*^o^ signatures and its spatial variability, four different study domains have been selected, which have areas of: 30×30 km^2^, 5×5 km^2^ and 1×1 km^2^. The 30×30 km^2^ and 5×5 km^2^ domains are covered by a mixture of grassland and wetland, while for the 1×1 km^2^ domain a grassland and wetland have been selected. The areas have been selected in such way that the 5×5 km^2^ domain is included in 30×30 km^2^ domain, and the 1×1 km^2^ domains are included in the 5×5 km^2^ domain as is shown in Figure 2.

**Figure 3.**
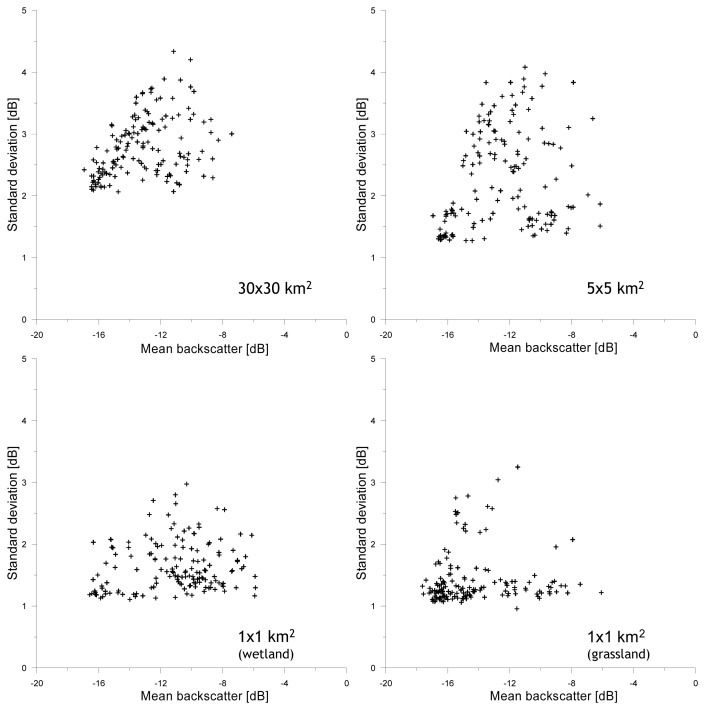
Standard deviation in σ^o^ observations plotted against the mean σ^o^ extracted from the ASAR WSM time series and four different spatial domains around Naqu station.

**Figure 4.**
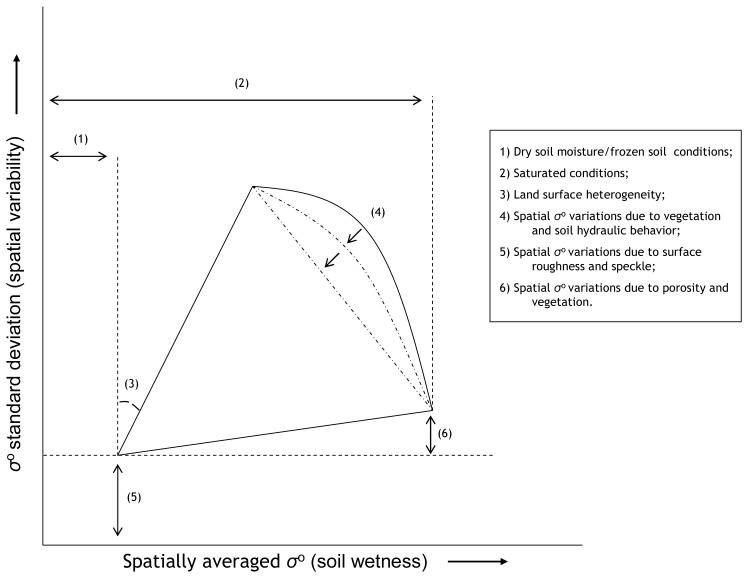
Schematization of the relationship between the mean *σ*^o^ and the standard deviation, and its coherence with specific land surface conditions and land surface characteristics (e.g. soil texture and heterogeneity).

Over each of the four domains *σ*^o^ observations have been averaged and the standard deviation (stdev) has been determined for all images within the ASAR WSM time series. The stdev is used, here, as a statistical variable representing the spatial *σ*^o^ variability. In Figure 3, the mean *σ*^o^ is plotted against the *σ*^o^ stdev for the four domains. The distribution of data points for each of the four spatial domains in figure 3 has a triangular shape. The explanation for this shape can be given as follows and has been schematized in Figure 4.

The lowest mean *σ*^o^ and *σ*^o^ stdev represent dry and frozen conditions. Because drought and freezing conditions have an impact on large areas, the spatial *σ*^o^ variability is small and is primarily influenced by speckle and spatial variations in surface roughness. Comparison of the minimum stdev's obtained from the different domains shows that the spatial *σ*^o^ variability increases with the size of the domain. This might be expected because over larger areas the variety of roughness conditions may be higher.

The mean *σ*^o^ value increases under conditions where liquid soil moisture is present. When thaw/freeze cycles and precipitation are homogeneously distributed throughout the study domains, the *σ*^o^ variability remains relatively low. The *σ*^o^ variability increases due to spatial differences in soil thermal and hydraulic properties, and precipitation inputs.

For each of the four domains, a well-defined and linear relationship exists between the mean *σ*^o^ and maximum stdev at specific *σ*^o^ levels and its slope could be seen as measure for the surface heterogeneity of a specific domain. Steeper slopes indicate a larger surface heterogeneity. For the 5×5 km^2^ domain, the slope is steepest and its surface heterogeneity may be considered to be the largest of the four domains. This is, however, also influenced by the distribution of wetlands and grasslands in the selected areas, because differences in land surface conditions between wetlands and grasslands persist, specifically during the monsoon. The similarity between the slopes in plots of the 30×30 km^2^, and 1×1 km^2^ wetland and grassland domains is striking and suggests a similarity in the surface heterogeneity between these areas. Additionally, it should be noticed that for the grassland domain the number of data points with a high stdev is small. Soils in the grasslands are sandy and have a high hydraulic conductivity. Over short time periods, soil moisture is transported from the top to deeper soil layers. Spatial variations due to dry-down cycle diminish, therefore, quickly and are difficult to capture by observations acquired at a 6-day temporal resolution.

During monsoon periods, land surfaces are wetter and vegetation grows, which both lead to an increase in the average *σ*^o^ values. Simultaneous to these higher *σ*^o^ observations, its spatial variability decreases for three reasons. Firstly, high *σ*^o^ values are only obtained when the entire domain is at or near saturation. Secondly, vegetation attenuates the soil surface scattering contribution and reduces the *σ*^o^ sensitivity to soil moisture changes. Thirdly, the *σ*^o^ response is less sensitive to soil moisture changes under wet than under dry conditions [23]. The decrease of the *σ*^o^ sensitivity to soil moisture changes due to either vegetation or soil wetness reduces the impact of spatial soil moisture variations on the *σ*^o^ variability.

Further, the plots show that the *σ*^o^ variability is higher under saturated than under dry conditions, which is caused by a combination of spatial variations in the porosity and vegetation. The increase in the spatial variability is stronger for larger domains (30×30 km^2^ and 5×5 km^2^) than the 1×1 km^2^ domains, which is expected because the variability in the porosity and vegetation tend to increase over larger distances.

The relationship between the mean *σ*^o^ and maximum stdev at specific *σ*^o^ levels under wet conditions is non-linear and is influenced by spatial variations in the soil hydraulic behavior and vegetation. Binding forces between the water molecules and soil particles determining the capillary force are, in general, smaller under wet than under dry conditions. A large amount of moisture is, therefore, transported relatively fast to deeper soil layers initiating the dry-down cycle. The time scale over which this process occurs depends strongly on the water retention capacity and hydraulic conductivity of the soil, which are both spatially variable. In addition, vegetation covering the soil surface reduces the *σ*^o^ sensitivity to soil moisture and destroys the spatial variability induced by the soil hydraulic behavior.

## 5. Comparison of spatial backscatter statistics with in-situ soil moisture measurements

**Figure 5.**
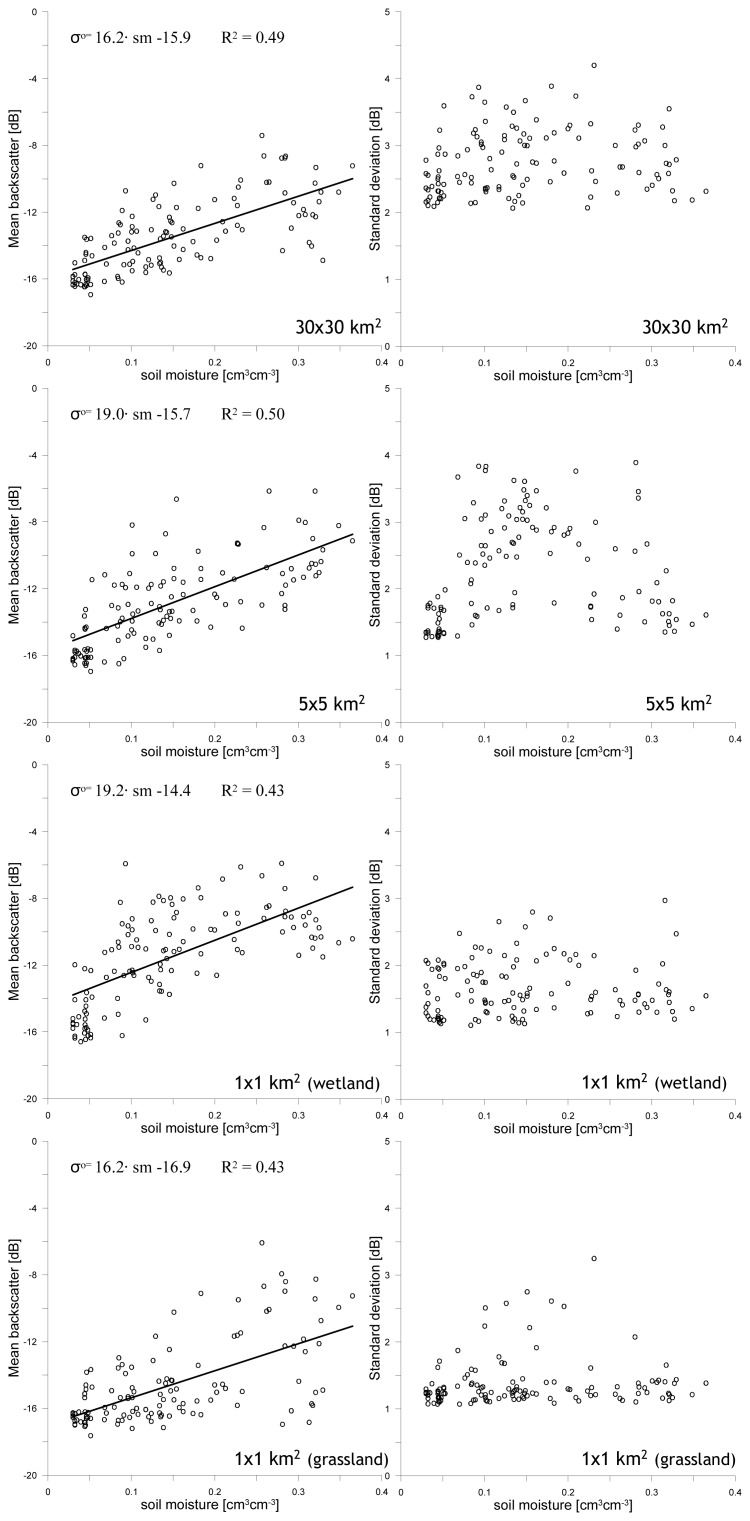
Comparison of the mean *σ*^o^ and stdev with soil moisture measured at Naqu station for the four selected spatial domain. The lines in the plots with the mean *σ*^o^ values represent linear regression functions of the form *σ^o^* = *a* · *sm* + *b*, where *sm* is the soil moisture content [cm^3^ cm^-3^].

In the previous section, the impact of land surface processes on the relationship between the mean *σ*^o^ and the stdev is described. Within this discussion the magnitude of the mean *σ*^o^ is implicitly assumed to be representative for the local soil moisture conditions and the *σ*^o^ stdev is utilized as indication for the spatial soil moisture variability. A robust validation of these assumptions would require intensive soil moisture sampling across the spatial domains over a long time period. Unfortunately, such data set is not available for the selected study area. However, at Naqu station an almost continuous time series of soil moisture measurements has been collected during the period in which ASAR observations were collected. These soil moisture measurements are plotted against the mean *σ*^o^ and stdev for the four spatial domains, which is shown in Figure 5. Linear regressions functions of the form *σ^o^* = *a* · *soil moisture*+ *b* have been computed and are presented in the plots. Statistics related to these regression functions are given in Table I.

It should be acknowledged for the comparison of mean *σ*^o^ and stdev with the measured soil moisture the SAR observations have not been corrected for the effects of surface roughness and vegetation. The objective of this investigation is, however, not to present (or apply) a methodology to correct the *σ*^o^ observations for the surface roughness and vegetation effects, but to analyze the relationship between the spatial backscatter statistics and in-situ soil moisture measurements. For the description (and application) of methodologies that correct *σ*^o^ observations for the effects of surface roughness and vegetation the reader is referred to previous investigations [i.e. 14-21].

Despite the mean *σ*^o^ is compared to soil moisture measured only at a single location and no correction for the effects of vegetation has been applied, still positive relationships are observed for all domains. Somewhat surprising is that for the 30×30 km^2^ and 5×5 km^2^ domains the coefficient of determination (R^2^) between the mean *σ*^o^ and soil moisture is higher than for the 1×1 km^2^ wetland and grassland domains. Because the size of the domains is smaller, the spatial soil moisture variability is smaller for the 1×1 km^2^ domains. Therefore, it would be expected that uncertainties due to imperfect representation spatial soil moisture variability are lower for the 1×1 km^2^ than for the 30×30 km^2^ and 5×5 km^2^ domains, which should result in better defined relationships between the mean *σ*^o^ and the measured soil moisture. Apparently, soil moisture dynamics measured at Naqu station is a better representation of the temporal soil moisture evolution observed over the larger 30×30 km^2^ and 5×5 km^2^ domains.

As is shown in Figure 2, Naqu station is located at the edge of a wetland. Therefore, soil moisture measured at this location will attain under dry conditions levels representative for grasslands, while under wet conditions soil moisture values will be higher due to the influence of the nearby wetland. These expected dynamics of the soil moisture measured at Naqu station can also be deduced from the distribution of the data points in Figure 5. For example, over 1×1 km^2^ grassland domain low mean *σ*^o^ values are observed even when the measured soil moisture is near saturation. Furthermore, the overall *σ*^o^ response observed over the 1×1 km^2^ grassland domain to the measured soil moisture is lower than for the other domains, while for the 1×1 km^2^ wetland domain the *σ*^o^ sensitivity to soil moisture is higher. This suggests that measured soil moisture at Naqu station is systematically higher than actual soil moisture in 1×1 km^2^ grassland domain and systematically lower than the actual soil moisture 1×1 km^2^ wetland domain. These observations supports that the soil moisture evolution measured at Naqu station represents better the soil moisture dynamics of the 30×30 km^2^ and 5×5 km^2^ areas, which explains the higher R^2^ for those domains.

The comparison of the *σ*^o^ stdev to the measured soil moisture for the four domains results in similar triangular distributions of the data points as is observed in Figure 3. The general explanation for the specific distribution of data points has been discussed in the previous section. The similarity in the triangular data point distributions between Figures 3 and 5 shows that the observed relationships between the mean *σ*^o^ and stdev can be considered to be representative for the temporally varying spatial soil moisture distributions in the selected domains.

However, also some differences are observed in the distribution of data points between Figures 3 and 5. For example, the relationship between the *σ*^o^ stdev and measured soil moisture towards dry conditions is not as well defined as relationship between the mean *σ*^o^ and the stdev in Figure 3. Moreover, several outliers are observed in Figure 5 deviating strongly from the general pattern. Explanation for these differences is that the *σ*^o^ stdev is compared in Figure 5 to soil moisture measured only at a single location. The measured soil moisture will, therefore, not always be representative for the spatial mean soil moisture of a spatial domain. In some cases, the measured soil moisture underestimates the actual conditions in a specific domain, while in other cases it overestimates the actual conditions. This is most obvious in the plot of the wetland domain. At a measured soil moisture of 0.03 (cm^3^cm^-3^), the *σ*^o^ stdev varies between 1.19 and 2.07 (dB). Since wetlands are to be systematically wetter than Naqu station (especially under those dry conditions), it can be expected that the actual soil moisture condition in the wetlands are wetter for the data points with a high stdev.

## 6. Conclusions and Discussion

In this paper, the spatial mean *σ*^o^ and stdev of four spatial domains obtained from a time series of 152 ASAR WSM images acquired over the Naqu river basin have been analyzed. The selected spatial domains have areas of 30×30, 5×5 km^2^ and 1×1 km^2^ (two domains). The 30×30 km^2^ and 5×5 km^2^ domains are covered by a mixture of grasslands and wetlands, while for the 1×1 km^2^ domain a grassland and wetland have been selected. Comparison of the mean *σ*^o^ values with the stdev values results in very specific triangular data point distributions for all spatial domains. The decrease of *σ*^o^ stdev as the mean *σ*^o^ decreases is observed because dry and freezing conditions have an impact on large areas and are, typically, homogeneously distributed across spatial domains. During the monsoon, however, intensive rain showers may saturate large areas. A decrease in the *σ*^o^ stdev is, therefore, observed as the mean *σ*^o^ increases. This impact of the soil moisture dynamics during monsoon and winter periods on the *σ*^o^ signature and its spatial variability is consistently observed for all four selected spatial domains. These findings are confirmed through a comparison of the mean *σ*^o^ and stdev with in-situ soil moisture measurements at a single station.

A consequence of the reported results is that the relationship between mean soil moisture and the spatial variability is not uniquely defined over the Tibetan Plateau and varies during the monsoon and winter periods. In downscaling coarse resolution soil moisture products changes in the relationship between the mean soil moisture and spatial variability should be considered, and should ideally be based on additional near real time data sources. SAR observations could be utilized to provide soil moisture information within passive microwave and scatterometer footprints.

## Figures and Tables

**Table 1.** Statistics related to linear regression functions computed between mean backscatter and soil moisture for the four spatial domains.

*Domain*	*a [dB/cm^3^cm^-3^]*	*b [dB]*	*R^2^ [-]*
30×30 km^2^	16.2	-15.9	0.49
5×5 km^2^	19.0	-15.7	0.50
1×1 km^2^ wetland	19.2	-14.4	0.43
1×1km^2^ grassland	16.2	-16.9	0.37
